# Anxiety and Depression in Patients with Prostate Cancer, at Cancer Diagnosis and after a One-Year Follow-Up

**DOI:** 10.3390/ijerph19159122

**Published:** 2022-07-26

**Authors:** Vítor Duarte, Natália Araújo, Catarina Lopes, Adriana Costa, Augusto Ferreira, Filipa Carneiro, Jorge Oliveira, Isaac Braga, Samantha Morais, Luís Pacheco-Figueiredo, Luis Ruano, Vítor Tedim Cruz, Susana Pereira, Nuno Lunet

**Affiliations:** 1 EPI Unit-Instituto de Saúde Pública, Universidade do Porto, Rua das Taipas, 135, 4050-600 Porto, Portugal; up201908722@edu.med.up.pt (V.D.); natalia.araujo@ispup.up.pt (N.A.); catarina.lopes@ispup.up.pt (C.L.); adriana.costa@ispup.up.pt (A.C.); samantha.morais@ispup.up.pt (S.M.); luis.ruano@ispup.up.pt (L.R.); vitor.tedimcruz@ulsm.min-saude.pt (V.T.C.); susana.pereira@ipoporto.min-saude.pt (S.P.); 2 Laboratório para a Investigação Integrativa e Translacional em Saúde Populacional (ITR), Rua das Taipas, 135, 4050-600 Porto, Portugal; 3 Departamento de Ciências da Saúde Pública e Forenses e Educação Médica, Faculdade de Medicina da Universidade do Porto, Alameda Professor Hernâni Monteiro, 4200-319 Porto, Portugal; 4 Instituto Português de Oncologia do Porto, Rua Dr. António Bernardino de Almeida, 4200-072 Porto, Portugal; augusto.carmo.ferreira@ipoporto.min-saude.pt (A.F.); ana.carneiro@ipoporto.min-saude.pt (F.C.); jorge.oliveira@cuf.pt (J.O.); isaac.braga@ipoporto.min-saude.pt (I.B.); 5 Instituto de Investigação em Ciências da Vida e Saúde, Escola de Medicina da Universidade do Minho, Campus de Gualtar, 4710-057 Braga, Portugal; lfigueiredo@med.uminho.pt; 6 Centro Hospitalar de São João EPE, Alameda Professor Hernâni Monteiro, 4200-319 Porto, Portugal; 7 Centro Hospitalar de Entre Douro e Vouga, Rua Dr. Cândido de Pinho, 4520-211 Santa Maria da Feira, Portugal; 8 Serviço de Neurologia, Hospital Pedro Hispano, Unidade Local de Saúde de Matosinhos, Rua Dr. Eduardo Torres, 4464-513 Senhora da Hora, Portugal

**Keywords:** prostate cancer, anxiety, depression, prevalence, prospective study

## Abstract

Prostate cancer (PCa) is the most prevalent among men, and psychological symptoms may affect many patients. This study aims to describe the prevalence of probable anxiety and depression before PCa treatments and after one year and to identify sociodemographic and clinical factors associated with these outcomes. Between February 2018 and March 2020, 292 patients recently diagnosed with PCa were recruited at the Instituto Português de Oncologia—Porto. The Hospital Anxiety and Depression Scale (HADS) was used to define probable anxiety and depression (cutoff = 11). The prevalence of probable anxiety remained stable from baseline to one year (7.8% vs. 8.5%, *p* = 0.866) while there was an increase in probable depression (3.1% vs. 6.8%, *p* = 0.012). After one year, probable depression persisted in 55.6% of patients with probable depression at baseline and 47.8% of those with probable anxiety at the first assessment had normal anxiety scores. At baseline, anxiety was more frequent among dwellers in rural areas (adjusted odds ratio—aOR, 95%CI: 2.80, 0.91–8.58) and less frequent in patients with body mass index 25–29.9 kg/m^2^ (aOR, 95%CI: 0.33, 0.12–0.91) compared to 18.5–24.9 Kg/m^2^, while those living alone had higher odds of depression (aOR, 95%CI: 6.35, 1.43–28.30). The frequency of anxiety and depression fluctuated during the course of treatment. Monitoring these symptoms would identify the most affected patients, contributing for a better use of mental health services.

## 1. Introduction

Prostate cancer is among the most prevalent cancers due to its high incidence and survival, emphasizing the importance of managing the burden of non-fatal outcomes on the overall health of patients. Worldwide, it is the second most incident cancer among men [1] and is projected to be the first after 2035 [2]. According to CONCORD [3], age-standardized five-year survival rose in most countries in the last decades; among patients diagnosed between 1995 and 2014, figures are in the range of 70–100%, which is largely attributed to early detection [3]. In Portugal, the five-year relative survival surpasses 90%, which is above the average estimate in Europe [4] and reflects the early detection of a large proportion of cases [5]. Indeed, despite there being no organized prostate cancer screening in Portugal, a recent study reported a prevalence of prostate antigen testing and/or digital rectal exam screening of 44.1% among men aged 40–79 years, whose main motivations were wanting to know if they have the disease, seeking for earlier detection, and for more effective treatments, whereas anxiety while waiting for the results was frequently reported (55.1%) as an adverse effect [6]. The high survival rate also reflects timely access to effective treatments. Indeed, the adoption of international guidelines for diagnosis, staging, treatment, and clinical follow-up, as well as the implementation of a multidisciplinary tumor board for treatment decision and planning, improve the delivery of the best available treatment for each patient with his involvement in sharing the decisions. Portuguese urologists have reported recommending active surveillance, whenever it is applicable, although patients still lag to adhere and choose active treatment [7]. In Portugal, patients with localized prostate cancer chose more frequently external beam radiation or radiotherapy than radical prostatectomy [8], whereas, in other countries where treatments are not paid for by the health system but by insurance, radical prostatectomy may be more frequently chosen. Anxiety and depression, in addition to being reported as the most common psychological disturbances among patients with cancer, are associated with poorer treatment outcomes, increased hospitalization, higher mortality rates, and lower treatment adherence [9,10,11,12,13,14]. Previous studies have estimated the prevalence of clinically meaningful anxiety and depressive symptoms, before, during, and after treatment, to be 27.04%, 15.09%, and 18.49%, respectively, for anxiety symptoms, and 17.27%, 14.70%, and 18.44%, respectively, for depressive symptoms, with these values being higher than in the general population [15]. Psychological health problems have a serious negative impact on the quality of life of prostate cancer survivors [16], upholding paramount importance on the overall adjustment process of the individuals, with fluctuating frequencies during treatment [17,18,19]. 

In addition to the stressful experience of cancer diagnosis, patients with prostate cancer may also be more at risk of depression due to advanced age at diagnosis, which has been associated with higher levels of depressive symptoms in several European countries, namely in Portugal [20]. Moreover, unemployment, lack of structured time, financial distress, and lower education were associated with psychological distress in the general Portuguese population [21] and patients with prostate cancer may also frequently present these risk factors, namely due to retirement, sick leave, and schooling not surpassing compulsory education, which was four years for most participants. On the other hand, hormone therapy has been associated with higher odds of depression, and active surveillance, compared to curative treatments, with more anxiety symptoms [15]. Lastly, physical symptoms and side effects of treatment, such as sexual dysfunction and urinary incontinence, family and social concerns, as well as the cancer pathophysiology itself may also contribute to depression in prostate cancer patients [22].

Although there is an increasing amount of research on anxiety and depression in patients with prostate cancer, the literature on these conditions during the course of treatment is methodologically heterogeneous, namely due to the use of a variety of screening tools and cut-offs, and differences in the characteristics of patients, namely regarding the type of treatment, cancer stage, and country, which may have also contributed to heterogeneous results [15]. Moreover, although the prevalence of clinically relevant anxiety and depression symptoms was estimated before, during, and after treatments, these results come from cross-sectional analyses, with longitudinal studies being scarce and with a small sample size [13], not allowing to follow the course of these psychological conditions over time, namely the proportions of recovery or chronic anxiety and depression. 

The aim of the present study is to describe the prevalence of depression and anxiety at cancer diagnosis and after a one-year follow-up, the persistence of these conditions, and to identify sociodemographic and clinical factors associated with these outcomes. The variation in anxiety and depressive scores will also be reported as they may reflect subtler and generalized overall changes. This study is based on the NEON-PC (neuro-oncological complications of prostate cancer) prospective study designed to evaluate cognitive decline among patients with prostate cancer. The participants were partially followed before and after the onset of the COVID-19 pandemic [23]. The fear of becoming infected with the SARS-CoV-2 virus and dying, and the social and physical restrictions imposed to control the pandemic can have consequences on the mental health of the general population [24], and, in cancer patients, the fear that the pandemic may affect cancer care with delays in staging exams and treatment procedures, as well as the distance in healthcare professional–patient relationships also contribute to the overall effect of the pandemic on the mental health of cancer patients [25,26]. Therefore, we describe the aforementioned psychological outcomes stratified by the time of the one-year assessment being before or after the onset of the COVID-19 pandemic in Portugal.

## 2. Methods

### 2.1. Setting and Participants

This prospective study was conducted at the Instituto Português de Oncologia do Porto (IPO-Porto). This is a public institution, one of the largest cancer hospitals in Portugal, which receives patients mainly from the northern region, after a referral from the family doctor or according to inter-hospital collaboration protocols. Possible treatments recommended in international guidelines are discussed within a multidisciplinary board and with the patient. 

**Figure 1:**
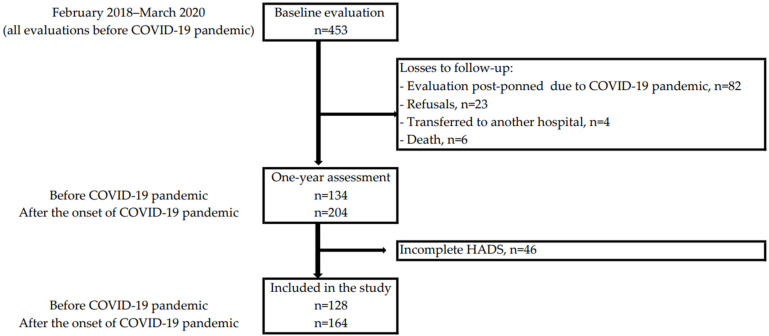
Flowchart describing the participation at each evaluation of the NEON-PC cohort and the participants included in the study.

This study is based on the NEON-PC project whose study protocol was previously described in detail [27]. Briefly, between February 2018 and March 2020, patients with a recent diagnosis of prostate cancer confirmed by biopsy were consecutively recruited. Eligibility and exclusion criteria were those established for the main outcome of the NEON-PC study—cognitive decline. Therefore, patients with less than one year of formal education and non-native Portuguese speakers were excluded, as well as those with a history of chemotherapy or radiotherapy for a previous cancer, those who already had started androgen deprivation therapy (ADT), and those with a psychiatric or neurologic condition impairing cognitive function identified by consultation of medical records or patients’ reports and confirmed by neurologist. Due to the COVID-19 pandemic, no evaluations were performed between 10 March and 30 June 2020. All participants (n = 453) had a baseline evaluation before the onset of the COVID-19 pandemic, and until May 2021 a total of 338 participants underwent the one-year evaluation; the follow-up took place before and after the first lockdown for 134 and 204 participants, respectively. Reasons for not performing the one-year evaluation (n = 115) were: evaluation postponed due to the COVID-19 pandemic (n = 82), refusal (n = 23), transfer to another hospital (n = 4), and death (n = 6). In addition to these losses, 46 participants did not complete the HADS at baseline and/or at one year and were not included in the present study (Figure 1). Those included (n = 292) and not included (n = 161) in data analysis were not significantly different regarding age (mean age, standard deviation, in years: 67.8, 7.2 vs. 67.8, 7.2; *p* = 0.989) or education (median, percentiles 25 and 75, in years: 4, 5 and 10 vs. 4, 4 and 8; *p* = 0.104).

### 2.2. The Hospital Anxiety and Depression Scale

The Hospital Anxiety and Depression Scale (HADS) was used to assess anxiety and depression symptoms. This self-administered questionnaire is one of the most frequently used in cancer patients [28] and has been validated in the Portuguese population, showing adequate reliability for the anxiety and depression subscales (Cronbach’s α of 0.76 and 0.81, respectively), 15-day test–retest correlations of 0.75 for anxiety scores and 0.75 for depression scores, and adequate content validity, suggesting that it measures the same constructs, in the same way, as the original HADS form [29]. The HADS is composed of 14 questions, each having four possible answers, graded from zero to three. Seven questions, collectively grouped as the HADS-D sub-scale, are the subcomponent that evaluates depressive manifestations, and the other seven, which evaluate anxiety, are grouped as the HADS-A subscale. According to the original article and to the validation study in the Portuguese population, participants were classified as having clinically significant anxiety and/or depression symptoms based on the HADS-A and HADS-D sub-scores (each ranging from zero to 21), respectively, if the corresponding sub-scores were equal to or higher than 11 [30]. Patients who had an HADS-A/HADS-D score equal to or higher than 11 at baseline, and below eight at the one-year evaluation, were considered to have recovered from anxiety/depression. If the HADS-A/HADS-D score was equal to or higher than 11 at both evaluations, anxiety/depression was considered stable over time.

All the baseline HADS evaluations and the follow-up assessments before the first COVID-19 lockdown were performed in person at IPO-Porto. After the lockdown, the follow-up assessments were completed at home and sent back by mail in a pre-paid envelope.

### 2.3. Sociodemographic Characteristics of the Participants and Clinical Information

A structured interview was conducted by a trained member of the research team to obtain the sociodemographic, lifestyle, and comorbidity characteristics of the patients.

Area of residence was classified according to national urban area typology guidelines (TIPAU 2014) [31], which stratifies parishes as predominantly urban, moderately urban, and predominantly rural. Alcohol consumption was classified considering the reference value of 20 g/day for men aged between 18 and 64 years, and 10 g/day for men aged 65 years or older [32,33]. Physical activity was classified according to the reference value of 150 min/week of moderate physical activity, 75 min per week of vigorous physical activity or the equivalent amount of time of a combination of moderate and vigorous physical activities [(minutes of vigorous physical activity per week × 2 + minutes of moderate physical activity per week) ≥ 150 min] [34]. Information on comorbidities (chronic health problems other than a psychiatric or neurologic condition) was obtained by self-report and from clinical files. The Tumor, Nodes, Metastases classification of the American Joint Committee on Cancer was retrieved from medical records to classify prostate cancer stage before treatment [35], and two groups were considered according to nodes involvement and metastases: no suspicion of node positivity for cancer cells and no metastases, and lymph nodes positive for cancer and/or metastases. Treatments performed during the first year of follow-up were classified into three groups: active surveillance, treatments with curative intent (brachytherapy, radical prostatectomy, and external beam radiation with or without ADT), and treatments with palliative intent (ADT with or without chemotherapy). The recruitment and baseline evaluations were performed after the patients had received the proposal for treatment.

### 2.4. Data Analysis

Patients’ characteristics and clinical variables were described using absolute and relative frequency. HADS scores at baseline and at one year were compared with the Wilcoxon test for paired data, and the McNemar’s test was used to compare the prevalence of anxiety and depression at the two evaluations. The Wilcoxon test for independent groups and the chi-square test were used to compare HADS scores and the prevalence of anxiety and depression, respectively, between patients with the one-year evaluation performed before or after the onset of the COVID-19 pandemic (first case reported in Portugal on 2 March 2020).

Participants with an HADS-A/HADS-D score below 11 at baseline and equal to or higher than 11 at one year were considered incident cases of anxiety/depression. Logistic regression was used to compute crude odds ratios (ORs), with the corresponding 95% CI for the association between patients’ characteristics and anxiety or depression outcomes. Potential confounders of the association between patients’ and tumor-related characteristics with anxiety/depression were identified in the literature. Therefore, age [36,37] and education [38] were included in the multivariate logistic models to estimate OR adjusted for these confounders (aOR).

Considering the main objective of estimating the frequency of clinically significant anxiety/depression symptoms over time, a sample of 289 participants allows to estimate proportions up to 25% with a 95% confidence interval (CI) up to 10% wide. This sample size also allows to detect significant associations with OR ≥ 3.5 or ≥3 between patients or tumor-related factors with a prevalence of at least 20%, (e.g., type II diabetes) or at least 50%, respectively, and clinically significant anxiety/depression symptoms, considering a 5% level of significance and a power of 80%. All analyses were performed using STATA v.15 (StataCorp, College Station, TX, USA). All tests were two-sided and a *p* < 0.05 was considered significant.

### 2.5. Ethical Approval

Ethical approval was obtained from the Ethics Committee of the Portuguese Institute of Oncology of Porto (Ref. CES 89/017) and by the Portuguese Data Protection Authority (Authorization 3478/2017). The study was performed in accordance with the ethical standards as laid down in the 1964 Declaration of Helsinki and its later amendments. Written informed consent was obtained from all participants after the project’s aims and procedures had been fully explained by a member of the research team.

## 3. Results

### 3.1. Description of Participants

The sociodemographic, lifestyle and clinical characteristics of participants are presented in Table 1. The mean age was 67.8 years (standard deviation, 7.2) and the mean education was 7.6 years (standard deviation, 5.0).

A total of 8.0% lived alone and 10.7% in predominantly rural areas, and more than 80% had no lymph node or distant metastasis and underwent treatment with curative intent. Less than one in every ten were current smokers and more than half reported less than 150 min of physical activity per week. Participants evaluated one year after the onset of the COVID-19 pandemic had a higher level of education and performed prostate cancer treatments with palliative intent less frequently.

### 3.2. Variation of Anxiety and Depression over Time

**Figure 2:**
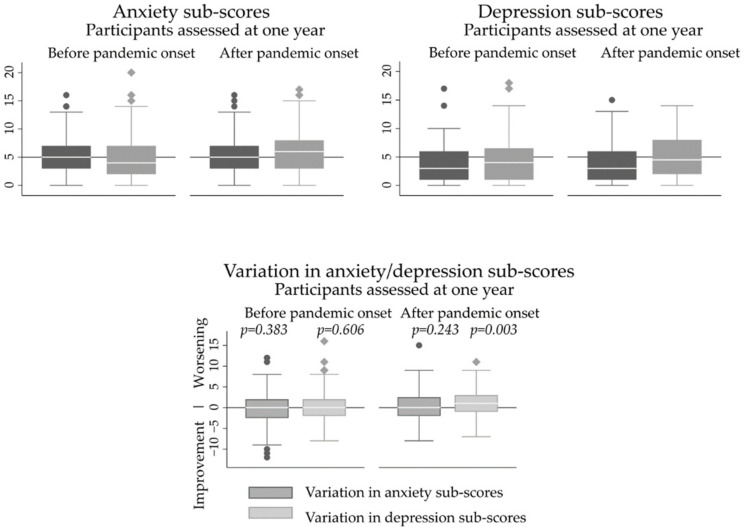
Distribution of anxiety and depression sub scores of the Hospital Anxiety and Depression Scale, at baseline and after one year, and of the variation in sub scores from baseline to the one-year evaluation, according to the period in which the one-year evaluation was performed (before or after the onset of COVID-19 pandemic). *p* values refer to testing the null hypothesis of the variation in scores being zero.

Figure 2 depicts the variations of anxiety and depression scores according to whether the one-year assessment was carried out before or after the onset of the COVID-19 pandemic. A statistically significant increase in depression scores (median (P25,P75): 3 (1,6) vs. 4.5 (2,8); *p* = 0.003) was observed during the first year of follow-up among participants who performed the one-year assessment after the onset of the pandemic, but the increase was not statistically significant when considering participants with both assessments performed before COVID-19 pandemic (median (P25,P75): 3 (1,6) vs. 4 (1,6.5); *p* = 0.243). For the variation in anxiety scores over time, no statistically significant differences were observed, independently of the timing of the one-year evaluation (median (P25,P75): 5 (3,7) vs. 4 (2,7), *p* = 0.606 and 5 (3,7) vs. 6 (3,8), *p* = 0.383, when the one-year evaluation was performed before or after the onset of the pandemic, respectively). Decreases (improvement) greater than two points in the anxiety and in the depression scales were observed in 22.9% and 17.8% of participants, respectively, whereas increases (worsening) larger than two points were observed in 23.3% and 24.3% of the participants in the anxiety and depression scales, respectively. No statistically significant differences were observed between values before or after the onset of the pandemic, although there was a marginally significant higher proportion of participants with decreases >2 in depressive scores (improvement) before than after the onset of the pandemic (22.7% vs. 14.0%, *p* = 0.056). Among participants with normal anxiety scores at both evaluations, 16.7% had an increase of at least three points after one year, being this value of 13.7% regarding the depression scale.

Anxiety and depression indicators at baseline and at the one-year follow-up assessments are presented in Table 2. The prevalence of clinically relevant anxiety symptoms remained stable over time (7.8% and 8.5% at baseline and at one year, respectively, *p* = 0.866) while there was an increase in clinically significant depressive symptoms after one year (3.1% vs. 6.8%, *p* = 0.012); the latter was more pronounced among patients with baseline and follow-up evaluations before the onset of the pandemic.

There were 7.0% and 5.3% incident cases of clinically significant anxiety and depression at the one-year evaluation, respectively. The group who had the follow-up evaluation after the onset of the COVID-19 pandemic had fewer incident cases of clinically meaningful anxiety (6.0% vs. 7.5%, *p* = 0.634) and depression (3.8% vs. 7.1%, *p* = 0.215) compared to patients who had assessments before the pandemic, but differences were not statistically significant.

Between baseline and follow-up, a total of 3.8% and 0.3% of the entire cohort were patients who recovered from clinically significant anxiety and depression symptomatology, respectively, corresponding to 47.8% and 11.1% of the patients who had clinically significant anxiety and depression symptoms at baseline, respectively. The proportions of the cohort with clinically significant anxiety and depression both at baseline and after one year were 2.0%% and 1.7%%, respectively, corresponding to the persistence of clinically significant symptomatology in 26.1% and 55.6% of those who also presented these levels of symptoms at baseline. Among those who had clinically significant anxiety at baseline, recovery was less frequent after the onset of the COVID-19 pandemic (33.3% vs. 75.0%, *p* = 0.057).

All participants with clinically significant anxiety and/or depression symptoms at baseline except four, who could not be contacted before the one-year evaluation, were asked if they wanted to be referred to the psycho-oncological department at IPO-Porto. Among those with anxiety (n = 23) at baseline, 34.7% were not interested in clinical follow-up and all had normal scores at one year except one with borderline score. Among those who accepted psychological care, half still had high levels (≥11) of anxiety after one year. Of the participants with HADS-D scores ≥11 at baseline (n = 9), 22.2% refused the referral to psychological follow-up, of whom, half had high levels of depression at one year, and 80.0% of those who accepted the referral had HADS-D scores ≥11 after one year.

### 3.3. Factors Associated with Anxiety and Depression

**Figure 3:**
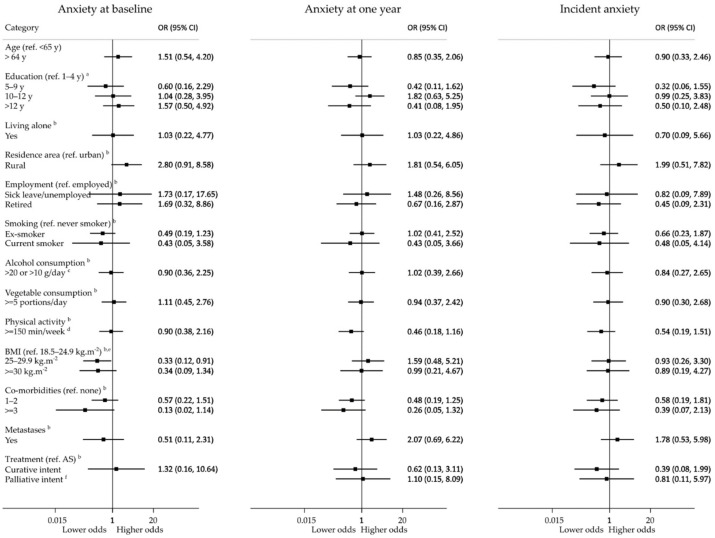
Associations between baseline variables and anxiety during the first year after prostate cancer diagnosis; AS, active surveillance; BMI, body mass index; ^a^ adjusted for age; ^b^ adjusted for age and education; ^c^ >20 g/day for men aged 18–64 years and >10 g/day for men aged 65 or older; ^d^ at least 150 min of physical activity weekly (sum of minutes of moderate physical activity + 2 × minutes of vigorous physical activity); ^e^ no participants had a BMI below 18.5 kg/m^2^; ^f^ no participants had the outcome.

**Figure 4:**
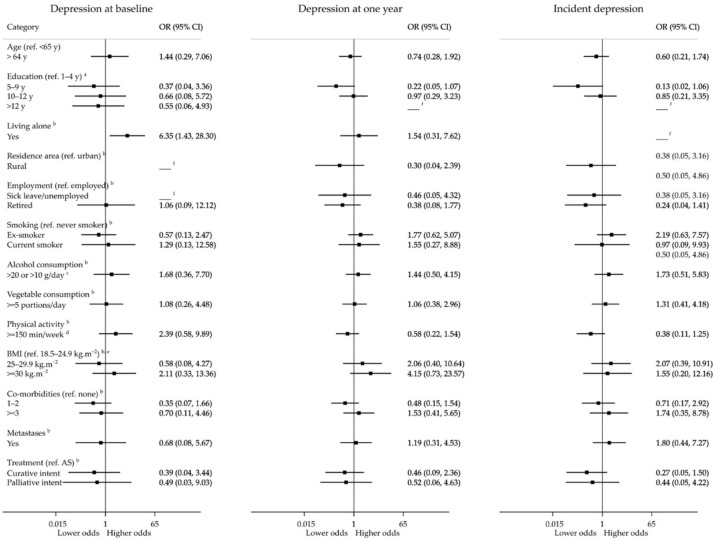
Associations between baseline variables and depression during the first year after prostate cancer diagnosis. AS, active surveillance; BMI, body mass index; ^a^ adjusted for age; ^b^ adjusted for age and education; ^c^ >20 g/day for men aged 18–64 years and >10 g/day for men aged 65 or older; ^d^ at least 150 min of physical activity weekly (sum of minutes of moderate physical activity + 2 × minutes of vigorous physical activity); ^e^ no participants had a BMI below 18.5 kg/m^2^; ^f^ no participants had the outcome.

Figure 3 and Figure 4 depict the association between different patients’ baseline characteristics and anxiety and depression. Participants living in predominantly rural areas were more likely to present relevant anxiety symptoms (aOR, 95% CI: 2.80, 0.91–8.58) before treatments. Those with a body mass index (BMI) between 25 and 29.9 kg/m^2^ had lower odds of anxiety at baseline (aOR, 95% CI: 0.33, 0.12–0.91) than those with a BMI between 18.5 and 24.9 kg/m^2^, but not at follow-up (aOR, 95% CI: 1.59, 0.48–5.21). Living alone was associated with higher odds of clinically relevant depression at baseline (aOR, 95% CI: 6.35, 1.43–28.30), but not at follow-up (aOR, 95% CI: 1.54, 0.31–7.62). None of the participants living alone had an onset of clinically relevant depressive symptoms during the one-year follow-up. Higher levels of education, comorbidities, more advanced cancer stage, and curative and palliative treatments were associated with lower odds of depression at baseline, although these differences were not statistically significant. No statistically significant associations were observed for the incidence of clinically relevant depression.

For participants evaluated at one year before the COVID-19 pandemic, no prevalent or incident cases of anxiety or depression in the active surveillance group were observed, whereas for those whose one-year assessment occurred after the onset of the pandemic, incident cases of anxiety were more frequent in the active surveillance group (15.4%, n = 2) than in curative (5.6%, n = 7) and palliative (0%, n = 0) treatment groups (*p* = 0.260), as well as incident cases of depression which were 15.4% (n = 2), 3.0% (n = 4) and 0% (n = 0), respectively (*p* = 0.07).

## 4. Discussion

This study provides contemporary data on depression and anxiety among patients with prostate cancer, at diagnosis and after one year. Increases (worsening) of at least three points in anxiety scores were observed in nearly a quarter of participants, and also regarding depression symptoms, in nearly a quarter, whereas decreases (improvement) of at least three points in scores were noticed in less than a quarter of participants regarding the anxiety scale and less than a fifth, regarding the depression scale. The prevalence of anxiety remained stable and close to 8% during this period, although the one-year cumulative incidence was 7% and almost half the patients with anxiety at baseline had normal scores after one year. The prevalence of depression more than doubled after one year, although remaining below 7%, the one-year cumulative incidence was 5.3%, and only 11% of prevalent cases at baseline had normal scores after one year.

Among participants with normal anxiety/depression scores at both evaluations, one in seven and one in eight participants had an increase in anxiety and depression scores, respectively, of at least three points after one year, which may be considered clinically meaningful, taking into account the three points interval which defines borderline cases of anxiety/depression, and the two points difference for the minimal clinically important difference reported in several studies using the HADS in patients with respiratory diseases [39,40,41]. These results suggest that the mental health of patients with prostate cancer worsens in a larger proportion of participants during the first year after the cancer diagnosis than what incidence data reveal.

The prevalence of significant anxiety and depression symptoms was lower than in previous evidence syntheses. In 2014, Watts, et al. [13] reported the prevalence of anxiety and depression symptoms across all phases of treatment (HADS cut-off ≥ 8) between 15% and 27%, and in 2020, Brunckhorst, et al. [15] reported a prevalence of 16.86% and 17.07% for anxiety and depressive symptoms, respectively, without specifying the HADS cut-off used, or treatment phase considered. In the former, studies included in the systematic review were conducted in Northern Europe countries, Australia, Canada, and the USA, before 2010. In the latter, studies published until 2019 were included and the prevalence of depression symptoms was lower in Asian than in North American studies. Indeed, changes in screening rates and treatments over time and among different cultures, namely uptake of active surveillance, may explain the differences in the results of the present study. At IPO-Porto, treatments are discussed within a multidisciplinary tumor board of oncologists, urologists, radiation therapy doctors, and imaging specialists to identify the best possible treatments according to international guidelines and to present the available options to the patient, who may choose, if applicable, between different treatments. Differences in the uptake of more aggressive treatment may exist among high-income countries, depending on the access to private health insurance, and patients with higher incomes opt more frequently for surgery. In addition to differences in the uptake of treatments over time and at different locations, heterogeneity in prevalence data may also be explained by the method used to detect depression. Studies using the HADS yielded a lower prevalence of depression than when other questionnaires were used, and the pooled prevalence of clinically diagnosed depression obtained in a meta-analysis was 5.81%, which is a closer value to the present result [15]. These comparisons must consider that the HADS, while being a valid screening tool used to track both borderline manifestations and clinically compatible diagnosis of anxiety and depression in various settings, can overestimate the prevalence of depressive and anxious disorders in comparison with clinical diagnosis, particularly when using the borderline cut-offs (≥8 points) [42,43].

Regarding the variability of anxiety and depression over time, Sharpley, et al. [19] compared cross-sectional data of men with prostate cancer, grouped according to time since cancer diagnosis (twelve periods of three months). There were no significant differences in anxiety and depression prevalence between groups, irrespective of treatments. The highest prevalence was observed in the initial period of receiving prostate cancer diagnosis (identified as the worst aspect of treatment in a later survey by the same author [44]). The prevalence in the active treatment period was lower and increased around the 10-month period, which corresponds to a period of re-evaluation of prostate cancer treatment. Sociodemographic and clinical differences between the groups evaluated in different moments over time may explain the lower values for anxiety and depression for the 10–12-month period after a cancer diagnosis than for the 0–3-month period, while the current study reported an increase in the prevalence of depression after one year of follow-up. Similarly, an increase in the prevalence of depression one year after cancer diagnosis was observed in a prospective study with patients mostly with early stage breast cancer evaluated with the HADS (cut-off ≥ 11) [45], although values were higher (8.1% and 13.6%), and a decrease in anxiety symptoms (38.0% to 24.6%) was observed. Gender differences, the type of cancer, and associated treatments may explain the differences in results.

Regarding the higher odds of anxiety among dwellers in rural areas in comparison to those who live in urban areas, there is literature supporting both, worse outcomes [46,47,48], and no significant differences [49,50,51,52], regarding mental health and quality of life in rural–localized patients in comparison to those in urban areas. This disparity in the literature can be attributed to the regional specificities of each cancer care network and health system arrangement, which directly organizes access, quality, and provision of care, and the resulting outcomes [53]. The further improvement of anxiety scores among patients one year after the baseline evaluation could be related to other factors: a German study assessing differences between cancer assistance in urban and rural environments (settings) singled out the patient–doctor relationship as the strongest predictor of mental health outcomes [50].

In our study, individuals in the normal BMI range group (18–25 kg/m^2^) had higher odds of presenting anxiety symptoms at the initial phase of the treatment when compared to overweight patients. Although weight loss and lack of appetite are expected as part of the general symptomatology overlapping both cancer and the most prevalent mental disorders, such as depression and anxiety [10], which can partially explain the findings, there is a scarcity of studies scrutinizing the possible relation between weight profiles and anxiety at the initial stages of treatment. A study in the United States found no significant relationship between BMI group and mental health-related quality of life in prostate cancer survivors [54]. Another study found significant psychological distress in overweight and obese cancer survivors who reported the need for dietary support [55]. A study among patients with cancer before receiving chemotherapy found a significant association between higher BMI scores and anxiety [56].

Regarding depression, living alone was associated with more depressive symptoms at baseline, but not at one year. Social isolation is associated with poor coping skills and a lack of social support. Enforced isolation due to COVID-19 lockdown procedures could have worsened the patients’ coping skills. Patients with prostate cancer appear to be at an increased risk of depression during COVID-19, partially aggravated due to feelings of isolation [57]. In Portugal, older individuals who lived alone, with perceived low social support, and who did not engage in leisure activities were more vulnerable to depression [58].

Compared with a previous cross-sectional study [59], the overall anxiety prevalence in the present work were higher than in the Portuguese elderly male population (7.8% and 8.5% vs. 4.6%) and the depression prevalence was lower (3.1% and 6.8% vs. 8.4%), although differences may not be significant considering the confidence interval of the estimates. Our results for the prevalence of depression seem to be similar to those obtained in a meta-analysis of 53 studies using the HADS-D cut-off 11, which yielded a pooled mean value of 8% (95% CI  =  7–9%), in the overall cancer population, and showed that the prevalence of depression (HADS-D cut-off eight or the Center for Epidemiologic Studies cut-off sixteen) was lower in patients with cancers of the male genitalia and urinary tract (up to 17%) than in those with cancers of the female genitalia, digestive tract and bone and soft tissue (up to 33%) [60].

This study adds to the available evidence on this topic a standardized prospective evaluation of anxiety and depression symptoms among patients with prostate cancer since their diagnosis, along with a detailed description of baseline and follow-up indicators of anxiety and depression. The longitudinal design of this study allowed for describing the persistence and recovery from high levels of anxiety and depression symptomatology over a one-year period. The course of anxiety and depression has been reported but regarding the variation in mean scores and not the classification of clinically significant symptoms [61,62], which is more interpretable information for clinical practice and for the planning of resources to manage clinically significant psychological problems.

However, some limitations need to be acknowledged. The single hospital design of this study may limit external validity, although the aforementioned institution is one of the largest hospitals and the regional referral center in the northern region of the country, which should minimize this limitation. We used the HADS to assess anxiety and depression, which would need to be confirmed with a clinical diagnosis. However, nearly half of the participants with clinically significant anxiety and/or depression identified with the HADS at any evaluation refused to be referred for a psycho-oncological consultation at IPO-Porto, most of them reported they did not feel the consultation was necessary. The disagreement between the HADS classification and seeking psychological help should be further studied. On the other hand, we did not collect data on psychological follow-up at IPO-Porto or at other health services. The number of participants in each treatment led to some of the groups (such as active surveillance) having a low number of individuals, which precluded stratified analyses by treatment. Larger cohort studies will contribute to the improvement of statistical power. Other patients’ characteristics, namely resilience and social support were not investigated in the present study and residual confounding may also exist in our analysis, as results were adjusted only for age and education.

## 5. Conclusions

The study shows the variability of anxiety and depression symptoms over the course of a one-year period since a recent diagnosis of prostate cancer, including incidence, recovery, and maintenance of mental status. Monitoring these symptoms will allow for the identification of the most affected patients, contributing to better use of mental health services.

## Figures and Tables

**Table 1:** Sociodemographic characteristics, lifestyles, and clinical information of the participants.

		Timing of the One-Year Evaluation in Relation to the COVID-19 Pandemic Onset		
	All	Before	After	
	n (%)	n (%)	n (%)	*p*
Age (years)				
<65	85 (29.1)	32 (25.0)	53 (32.3)	0.172
≥65	207 (70.9)	96 (75.0)	111 (67.7)	
Education (years)				0.051
1–4	145 (49.7)	74 (57.8)	71 (43.3)	
5–9	65 (22.3)	20 (15.6)	45 (27.4)	
10–12	37 (12.7)	15 (11.7)	22 (13.4)	
>12	45 (15.4)	19 (14.8)	26 (15.9)	
Living alone				0.678
No	263 (92.0)	114 (91.2)	149 (92.5)	
Yes	23 (8.0)	11 (8.8)	12 (7.5)	
Area of residence ^a^				0.961
Urban	241 (89.3)	108 (89.3)	133 (89.3)	
Rural	29 (10.7)	13 (10.7)	16 (10.7)	
Employment				0.475
Sick leave/unemployed	13 (4.6)	6 (4.8)	7 (4.4)	
Employed	72 (25.4)	27 (21.8)	45 (28.1)	
Retired	199 (70.1)	91 (73.4)	108 (67.5)	
Smoking status				0.217
Never smoker	127 (44.3)	53 (42.4)	74 (45.7)	
Ex-smoker	137 (47.7)	58 (46.4)	79 (48.8)	
Current smoker	23 (8.0)	14 (11.2)	9 (5.6)	
Alcohol consumption ^b^				0.558
≤10 or 20 g/day	153 (56.3)	64 (54.2)	89 (57.8)	
>10 or 20 g/day	119 (43.8)	54 (45.8)	65 (42.2)	
Vegetables consumption				0.063
<5 portions/day	198 (69.0)	79 (63.2)	119 (73.5)	
≥5 portions/day	89 (31.0)	46 (36.8)	43 (26.5)	
Physical activity ^c^				0.891
<150 min/week	161 (55.1)	70 (54.7)	91 (55.5)	
≥150 min/week	131 (44.9)	58 (45.3)	73 (44.5)	
Body mass index (kg/m^2^) ^d^				0.297
18.5–24.9	65 (26.1)	36 (30.5)	29 (22.1)	
25.0–29.9	133 (53.4)	58 (49.2)	75 (57.3)	
≥30	51 (20.5)	24 (20.3)	27 (20.6)	
Comorbidities				0.357
None	58 (19.9)	30 (23.4)	28 (17.1)	
1–2	185 (63.4)	76 (59.4)	109 (66.5)	
≥3	49 (16.8)	22 (17.2)	27 (16.5)	
Cancer stage				0.060
Any T, N0, M0	250 (85.6)	104 (81.3)	146 (89.0)	
Any T, N1 and/or M1	42 (14.4)	24 (18.8)	18 (11.0)	
Treatment				0.003
Active surveillance	15 (5.1)	1 (0.8)	14 (8.5)	
Curative intent	251 (86.0)	111 (86.7)	140 (85.4)	
Palliative intent	26 (8.9)	16 (12.5)	10 (6.1)	

^a^ Classification of the parish where the participant lives, according to urban–rural typology from the Instituto Nacional de Estatística (TIPAU 2014, version V03486, available at: https://smi.ine.pt/Versao/Detalhes/3486, accessed on 21 October 2021); ^b^ >20 g/day for men aged 18–64 years and >10 g/day for men aged 65 or older; ^c^ at least 150 minutes of physical activity weekly (minutes of moderate physical activity + 2 × minutes of vigorous physical activity); ^d^ no participants had a BMI below 18.5 kg/m^2^.

**Table 2:** Anxiety and depression states among patients with prostate cancer during the first year after cancer diagnosis, according to the time period, before or after COVID-19 pandemic onset, in which the one-year evaluation was performed.

	Anxiety	Depression
	% (95% CI)	% (95% CI)
**All (n = 292)**		
Prevalence at baseline	7.8 (5.0–11.5)	3.1 (1.4–5.7)
Prevalence at one year	8.5 (5.6–12.3)	6.8 (4.2–10.3)
One-year cumulative incidence ^a^	7.0 (4.3–10.7)	5.3 (3.0–8.5)
Stable anxiety/depression ^b^	2.0 (0.7–4.4)	1.7 (0.6–4.0)
Recovery from anxiety/depression ^c^	3.8 (1.9–6.6)	0.3 (0.0–1.9)
**Before COVID-19 pandemic onset (n = 128)**		
Prevalence at baseline	6.2 (2.7–11.9)	1.6 (0.2–5.5)
Prevalence at one year	7.8 (3.8–13.9)	8.6 (4.4–14.8)
One-year cumulative incidence ^a^	7.5 (3.5–13.8)	7.1 (3.3–13.1)
Stable anxiety/depression ^b^	0.8 (0.0–4.3)	1.6 (0.2–5.5) ^d^
Recovery from anxiety/depression ^c^	4.7 (1.7–9.9)	0 (0–2.8) ^d^
**After COVID-19 pandemic onset (n = 164)**		
Prevalence at baseline	9.1 (5.2–14.6)	4.3 (1.7–8.6)
Prevalence at one year	8.5 (4.7–13.9)	5.5 (2.5–10.2)
One-year cumulative incidence ^a^	6.0 (2.8–11.2)	3.8 (1.4–8.1)
Stable anxiety/depression ^b^	3.0 (1.0–7.0)	1.8 (0.4–5.3)
Recovery from anxiety/depression ^c^	3.0 (1.0–7.0)	0.6 (0.0–3.4)

CI, confidence interval; ^a^ participants with an anxiety/depression sub score lower than 11 at baseline, and with the anxiety/depression sub score at one-year equal to or higher than 11; ^b^ participants who presented clinically significant anxiety/depression at baseline and after one year (anxiety/depression sub scores at baseline and at one-year equal to or higher than 11); ^c^ participants with an anxiety/depression sub score equal to or higher than 11 at baseline, and with the anxiety/depression sub score at one-year lower than 8; ^d^ one-sided, 97.5% confidence interval.

## Data Availability

The datasets generated and analyzed in this study will not be publicly available given that the included patients do not specifically provide their consent for public sharing of their data and that anonymization is unlikely to be feasible, since the identification of patients treated in only one institution within a relatively short period may be possible when taking socio-demographic and clinical characteristics into account.
